# Effects of a *Bacillus licheniformis* Fermentation Extract and Monensin on the Rumen and Hindgut Microbiota Composition of Lactating Dairy Cows

**DOI:** 10.3390/ani15202980

**Published:** 2025-10-15

**Authors:** Phoebe Hartoonian, Lucille C. Jonas, Shedrack Omale, Sydney Rigert, Catherine Bradley, Erin Horst, Donald Beitz, Stephan Schmitz-Esser, Ranga Appuhamy

**Affiliations:** 1 Department of Animal Science, Iowa State University, Ames, IA 50011, USA; phoebeh@iastate.edu (P.H.); lcjonas@iastate.edu (L.C.J.); shedrack@iastate.edu (S.O.); slrigert@iastate.edu (S.R.);; 2 Interdepartmental Microbiology Graduate Program, Iowa State University, Ames, IA 50011, USA; 3 LandO’Lakes Inc., 100 Danforth Drive, Gray Summit, MO 63039, USA; 4 Elanco Animal Health, 2500 Innovation Way, Greenfield, IN 46140, USA

**Keywords:** *Bacteroidetes*, correlations, feed efficiency, feed additives, fermentation profile, *Firmicutes*, parity interactions

## Abstract

**Simple Summary:**

Monensin is a feed additive widely used to modify the rumen microbiota composition and thereby improve feed efficiency; meanwhile, new feed additives with similar effects are becoming available. Researchers speculate that feed additives designed to alter the ruminal microbiota can pass intact to the lower gut and influence the microbiota there as well. In this study, monensin and a new feed additive derived from the bacterium *Bacillus licheniformis* were added to the feed, alone and in combination, to determine if their effects on ruminal and hindgut microbiota, as well as feed efficiency, would be additive in dairy cows. The results, however, highlighted that using the *Bacillus licheniformis* fermentation extract alone—rather than in combination with monensin—might effectively improve feed efficiency by modulating the rumen microbiota composition rather than that of the lower gut. Monensin differentially modulated ruminal microbiota composition in primiparous and multiparous cows. Monensin increased milk fat content in multiparous cows but did not affect it in primiparous cows, in line with the rumen microbiota composition.

**Abstract:**

This research reports ruminal and fecal microbiota composition of lactating dairy cows enrolled in a study aimed at investigating the effects of a fermentation extract derived from *Bacillus licheniformis* (BLFE), monensin (Rumensin^®^; R), and their interactions on feed efficiency (FE, FE = milk yield/DMI). In a completely randomized design, 48 Holstein cows at 108 ± 35 days in milk were matched for parity and assigned to monensin (0 or 17.6 g/kg of DM) and BLFE (0 or 166 mg/kg of DM) in a 2 × 2 factorial arrangement. Treatments were fed daily for 63 d, including a 21 d adaptation period followed by a 42 d measurement period (P2). On d 38 and d 39 of P2, rumen-fluid (RF) and fecal samples were collected. DNA from RF and feces was sequenced using 16S rRNA gene-amplicon sequencing on an Illumina MiSeq platform. Fecal and RF volatile fatty acid (VFA) concentrations were analyzed, and propionate/acetate (P: A) was determined. The BLFE increased milk yield (3.3 kg/d) and FE (1.20 to 1.28), when fed alone rather than with monensin, while monensin increased energy-corrected milk yield (2.5 kg/d, *p* < 0.05), regardless of the BLFE in the diet. The BLFE tended to increase ruminal *Firmicutes*/*Bacteroidetes* (F: B) when fed alone, while alpha and beta diversities remained unmodified. The BLFE increased the abundances of *Bifidobacterium* (*p* = 0.02) and *Erysipelotrichaceae_*UCG-002 (*p* = 0.01) in RF, whereas monensin increased and decreased the abundances of *Oscillospirales_*ge (*p* = 0.02) and an unclassified *Clostridia* genus (*p* = 0.03), respectively. The monensin-suppressed *Clostridia* were negatively associated with ruminal P: A (r = −0.66; *p* < 0.01) and feed efficiency (r = −0.30; *p* = 0.04). The BLFE and monensin interactively affected several fecal genera (*p* < 0.05), but they had negligible or weak correlations with fecal P: A and FE. Overall, the results showed the ability of dietary supplementations of monensin and BLFE to increase milk production performance and FE by modulating ruminal rather than lower-gut microbiota composition, this is predominantly attributed to the ratio between the *Firmicutes* and *Bacteroidetes* abundances in lactating dairy cows.

## 1. Introduction

Ruminants possess a unique ability to convert plant biomass inedible to humans into highly nutritious food products, as a result of their symbiotic relationship with the rumen and lower-gut microbiota [1,2]. For instance, the ruminal microbiota digest and ferment plant fiber, producing volatile fatty acids (VFA), such as acetate (A) and propionate (P), which contribute up to 90% of the energy required by dairy cows to produce milk [3]. The ruminal microbiota composition dictates the ruminal VFA profile (e.g., P: A), which plays a crucial role in determining feed utilization efficiency (e.g., milk yield/feed intake) [4,5,6,7,8,9,10]. While the link between ruminal microbiota and feed efficiency has been studied widely, the connection between hindgut microbiota and feed efficiency has received less attention, possibly because of the limited role that hindgut fermentation plays in the supply of metabolizable energy in dairy cows. Nevertheless, Monteiro et al. [2] demonstrated that combined lower-gut and ruminal microbiota compositions yielded improved predictions of feed intake in dairy cows, compared to the use of ruminal microbiota alone (R^2^ = 0.80 vs. 0.35). These results highlight the importance of also investigating the involvement of lower-gut microbiota composition in modulating the production performance of livestock [11,12] in response to dietary interventions.

Among these dietary interventions, ionophores, such as monensin, have been widely used since the 1970s to improve gut health and feed efficiency in farm animals [13]. Monensin was first introduced to the poultry industry to control coccidiosis and was later approved (in 2004) for lactating dairy cows to improve milk production efficiency [13,14]. Monensin disrupts the Na^+^ and K^+^ ion gradient across the cell membrane that bacteria rely on for survival. Consequently, bacteria are forced to alter their cellular metabolism to expend a significant amount of energy in restoring the ion gradient, resulting in growth retardation and cell death [15,16]. It is widely believed that monensin can insert more effectively into the cell membranes of Gram-positive bacteria than those of Gram-negative bacteria, thereby altering the microbial balance and favoring the growth of Gram-negative bacteria [16,17]. By altering the microbiota composition in the rumen, monensin shifts the production of VFA to favor P over A, resulting in a more energy-efficient fermentation process and improved feed efficiency in dairy cows [13,14,15,16,17]. However, recent research emphasizes that the impact of monensin on Gram-positive, versus Gram-negative, bacteria is not always straightforward, and the varying sensitivities of bacteria to monensin depend on their cell wall composition and thickness, rather than their Gram-positive or Gram-negative status [18].

Given the current emphasis on improving feed efficiency and the sustainability of livestock, additional feed additives that favorably modify ruminal metabolism are being introduced [19]. Among these feed additives, live *Bacillus* species have shown promising results in cattle [19,20]. Chang et al. [21] demonstrated in vitro that live *Bacillus subtilis* increased ruminal P: A, indicating its potential to enhance feed efficiency to a level achieved with monensin. Another Gram-positive *Bacillus* species, *Bacillus licheniformis*, increased ruminal P: A to levels comparable with those achieved with monensin in sheep [22]. Additionally, feeding live *Bacillus licheniformis* enhanced microbial protein synthesis by 16–25% in both sheep and dairy cows [22,23], and feeding *Bacillus licheniformis*-derived enzymes increased feed efficiency by 8% in dairy cows [24]. These findings suggest that monensin and *Bacillus licheniformis* may enhance feed efficiency synergistically when fed together. Based on the literature showing the potential suppression of *Bacillus* species in the rumen by monensin [25], *Bacillus*-derived products (e.g., fermentation extracts) may be a better option than live cells for achieving additive feed-efficiency enhancement. Additionally, investigating how these feed additives interact to modify the composition of gastrointestinal microbiota may provide better insights into their effects on feed efficiency.. This study’s hypothesis was that feeding monensin and a fermentation extract derived from *Bacillus licheniformis* (BLFE) would additively improve milk production performance and feed in lactating dairy cows. The study’s objective was to investigate the individual and interactive effects of BLFE and monensin (Rumensin^®^; R), on production performance and feed efficiency. Additionally, the effects, particularly those of the interactions, on ruminal and lower-gut microbiota composition were examined for the first time to improve the current understanding, which is limited to findings on the individual effects of these feed additives [18,26,27]. While a separate publication comprehensively reports on the production and feed-efficiency effects [28], this report focuses on the impacts on ruminal and fecal microbiota composition.

## 2. Materials and Methods

### 2.1. Animals, Experimental Design, and Treatments

The animal trial protocol was approved by the Iowa State University (ISU) Animal Care and Use Committee (IACUC-22-203), and the trial was conducted at the ISU Dairy Teaching and Research Farm from December 2022 to March 2023. In a completely randomized experimental design, mid-lactating (DIM = 108 ± 35) Holstein cows (16 primiparous and 32 multiparous) matched for parity were assigned randomly to two doses [0 (–R) or 17.6 mg/kg of DM (+R)] of monensin (Rumensin^®^, Elanco Animal Health, Greenfield, IN) and two doses [0 (–BLFE) or 166 mg/kg of DM (+BLFE)] of BLFE (Purina Animal Nutrition LLC, Gray Summit, MO, USA), in a 2 × 2 factorial arrangement, which yielded four treatment groups (N = 12 cows per group), –R–BLFE, +R–BLFE, –R+BLFE, and +R+BLFE. Despite the targeted doses, the actual monensin and BLFE doses consumed by cows were 14 mg and 145 mg/kg of DM, respectively. This discrepancy was due to the observed DMI being higher than the anticipated DMI during the initial dose calculation.

Cows were housed in a free-stall pen equipped with Calan gates (American Calan Inc., Northwood, NH, USA) to measure individual cow feed intake. The baseline feed intake and milk production performance were recorded for 14 d, while feeding the regular total mixed ration (TMR) of the farm, containing monensin at 16 mg/kg of DM. Then, the monensin was removed from the TMR, and treatment doses were top-dressed on it for the next 63 d. Dhuyvetter [29] demonstrated that it took 21 d for the carry-over effects of monensin on milk production and feed efficiencies to disappear. Therefore, data were collected to determine the treatments’ effects from d 22 to d 63 (experimental period = 42 d). Cows were fed 110% of the previous day’s dry matter intake (DMI) once daily at 0700 h. Cows were milked twice daily, at 1000 and 2200 h, and yields were recorded electronically at each milking (Boumatic, Madison, WI, USA). Milk samples were collected from both milking sessions once every two weeks for milk composition analyses (CentralStar Cooperative, Kaukauna, WI, USA).

### 2.2. Rumen-Fluid and Feces Collection

Rumen-fluid and fecal samples for microbiome analysis were collected on d 39 and d 40 during the experimental period. Fecal samples were collected directly from the rectum, using sterile polyethylene gloves, at 0600 h. Approximately 400 g of feces were collected from the rectum, and a representative 10–15 g sample was transferred to sterile plastic bags; these samples were immediately frozen on dry ice. Another representative fecal sample (~100 g) was stored at −20 °C for VFA analysis. The rumen-fluid samples (~100 mL) were each collected into a clean Erlenmeyer flask using a clean oral stomach tube (Tygon^®^ tubing; 1.5 cm O.D. and 0.9 cm I.D) running through a speculum and attached to a hand-operated siphon pump, at 1400 h on d 60 and d 61 (24 cows per day). The flask and the tube were rinsed thoroughly with pressurized tap water between cows. Approximately 15 mL of rumen fluid was transferred from the flask into a conical tube, assessed visually for the presence of saliva, and immediately frozen on dry ice. Samples were retaken if saliva contamination was visible. It is noteworthy that the pH was not used as a measurement to monitor this contamination. Both the fecal and the rumen-fluid samples were stored at −80 °C until they were subjected to DNA extraction.

#### Rumen Fluid and Feces Volatile Fatty Acid (VFA) Analyses

The VFAs were analyzed according to the method in Wickramasighe et al. [30]. Thawed rumen-fluid and fecal samples were centrifuged at 4 °C and 9000× *g* for 15 min, and 5 mL of the supernatant was mixed with 1.0 mL of 25% metaphosphoric acid. The mixture was centrifuged for 10 min at 4000× *g* at room temperature, and the supernatant was separated. The acetate and propionate standards, each at dilutions of 1:1, 1:2, 1:4, and 1:6, were prepared using deionized water (Sigma-Aldrich, St. Louis, MO, USA). Then, 100 µL of 2-ethyl butyric acid (the internal standard) and the VFA standards were added to the supernatant of each rumen-fluid sample. The acetate and propionate concentrations of those samples were determined by using a Varian CP-3800 Gas Chromatograph with capillary columns (Varian Medical Systems, Palo Alto, CA, USA) in the Department of Animal Science at Iowa State University (Ames, IA, USA). The VFA concentrations in rumen or feces samples were determined based on the relationship between chromatography outputs and the standard concentrations established with regression analyses.

### 2.3. DNA Extraction, 16S rRNA Gene Amplicon Sequencing, and Bioinformatics Analysis

Following the thawing of samples in a water bath at 36 °C, DNA was extracted from approximately 0.25 g of each rumen-fluid and fecal sample by using the Qiagen DNeasy Powerlyzer Powersoil kit (Qiagen, Hilden, Germany) according to the manufacturer’s instructions, including the optional 4 °C incubation steps. Mechanical cell lysis was performed by using a Fisherbrand™ Bead Mill 24 Homogenizer (Fisher Scientific, Portsmouth, NH, USA), and DNA concentrations were measured by a Nanodrop 2000 spectrophotometer (ThermoFisher Scientific, Waltham, MA, USA). The DNA-extraction kit controls were prepared by using diethylpyrocarbonate (DEPC)-treated water and kit reagents, following the manufacturer’s instructions, to detect contamination during DNA quantification and isolation. After extraction, samples with a DNA concentration greater than 25 ng/µL were adjusted to 25 ng/µL using DEPC-treated water. This DNA concentration adjustment was required in order to achieve comparable concentrations across samples, which were required by the Iowa State University DNA facility (Ames, IA, USA), where the sequencing was conducted. Before 250 bp paired-end sequencing on an Illumina MiSeq Platform (Illumina, San Diego, CA, USA), PCR amplification of the 16S rRNA variable region V4 was conducted. For amplification, template DNA from each sample (one replicate per sample) was amplified by using Platinum Taq DNA Polymerase (Thermo Fisher Scientific, Waltham, MA, USA), with one replicate per sample, by using universal 16S rRNA gene bacterial primers [515F (5′-GTGYCAGCMGCCGCGGTAA-3′); and 806R (5′-GGACTACNVGGGTWTCTAAT-3′)] amplifying the variable region V4. All samples underwent PCR, with an initial denaturation step at 94 °C for 3 min, followed by 45 s of denaturing at 94 °C, 20 s of annealing at 50 °C, and 90 s of extension at 72 °C. This procedure was repeated for 35 total PCR cycles before a 10-min extension at 72 °C, followed by purification with a QIAquick 96 PCR Purification Kit (Qiagen, Hilden, Germany) according to the manufacturer’s instructions.

Sequence data were analyzed using the Mothur (v1.48.0) software according to the Mothur MiSeq Standard Operating Procedure and internal pipelines [31]. Raw paired-end reads were merged and filtered to generate contiguous sequences (contigs). Contigs were quality-filtered using the ‘make command.contigs’ command using a minimum read length of 250 bp, a zero ambiguities threshold, and a maximum homopolymeric region of 8 bp. Chimeric sequences were removed using the ‘chimera.vsearch’ command in Mothur, using the SILVA.gold reference database provided by the Mothur website. The remaining reads were aligned against the SILVA SSU NR database (V138) for alignment and taxonomic classification of operational taxonomic units (OTUs). Sequences were clustered into de novo OTUs with a 99% 16S rRNA sequence similarity threshold. The average sequencing depth was 60,302 sequences, with a standard deviation of 21,288 sequences. The data were imported from Mothur into R software (v4.3.1; R Core Team 2021) and processed using the phyloseq package in R (v1.34.0; [32]). The decontam package was used to identify and remove OTUs present due to contamination (v1.10.0; [33]). Several OTUs were classified as likely contaminants and removed from the original dataset of 230,994 OTUs, resulting in a total of 230,818 OTUs. Further, OTUs represented by fewer than 10 reads were removed to decrease the prevalence of spurious OTUs resulting from sequencing errors. Only 12,469 OTUs passed this quality-control step. The average reads value for the control samples was 7938, while the average reads value for the experimental samples was 60,302. Rumen-fluid and fecal samples were analyzed separately to estimate alpha and beta diversity parameters and the relative abundances of OTUs. Alpha diversity measures were subsampled to the lowest sample read number (32,287). Principal coordinate analysis (PCoA) plots were used to visually assess beta diversity. The PCoA plots were generated using Bray–Curtis distances [34]. All plotting was completed by using the R ggplot2 (v2_3.1.1; [35]) graphing package. OTUs were first classified by using the SILVA database (v138), and the representative nucleotide sequences of each OTU were then queried against the NCBI database to verify the taxonomic classification.

### 2.4. Calculations and Statistical Analyses

Feed efficiency was calculated by expressing milk yield (kg/d) as a ratio to DMI (kg/d), based on measurements taken during the last two weeks of the trial. The energy-corrected milk yield (ECM, kg/d) was calculated by using the following equation [36].

$$ECM\;=\;\left(12.82\;\times \;fat\;yield\right)\;+\;\left(7.13\;\times \;protein\;yield\right)\;+\;\left(0.323\;\times \;milk\;yield\right)$$

The P: A in rumen fluid and feces was calculated by expressing the molar percentages of propionate relative to the corresponding molar percentages of acetate. The ratios of *Firmicutes* to *Bacteroidetes* abundances (F: B) in rumen fluid and feces were calculated by expressing the relative abundance of *Firmicutes* relative to that of *Bacteroidetes.*

The treatments’ effects on alpha diversity, as well as the relative abundances of *Firmicutes* and *Bacteroidetes*, and F: B and P: A, were analyzed using the following statistical model, with the MIXED procedure of SAS software (v9.4; SAS Institute Inc., Cary, NC, USA).

Y_ilklm_ = µ + R_i_ + BLFE_j_ + E_k_ + P_l_ + (R × BLFE)_ij_ + C_ijklm_ + e_ijklm_

where Y = the response variable measurement, µ = overall mean, R = the fixed main effect of monensin, BLFE = the fixed main effect of BLFE, E = the fixed effect of the ecosystem (rumen fluid vs. feces), P_l_ = the fixed effect of parity, (R × BLFE) = the fixed effect of the interaction between R and BLFE, C = random effect of the cow, and e = residual error, assumed to be normally and uniformly distributed with a mean of zero. The interactions between dietary treatments and parity were tested, but were included in the final model only when significant (*p* < 0.05). The effects of the treatments on the most abundant genera, as well as the relative abundances of *Firmicutes* and *Bacteroidetes* and the F: B, in the rumen fluid and feces were analyzed separately by removing the E_k_ effect from the above model. The model without E_k_ was also used to analyze the effects of the treatments on production performance by including the baseline measurement as a covariate. The outliers were determined using Cook’s distance. The residual error distribution was evaluated using the Q–Q plots. The log transformation was applied to the response variable if the residual error distributions deviated from the assumed normality. The Pearson correlation coefficients (r) and corresponding *p*-values for relationships among the relative abundances of microbiota, feed efficiency, and P: A were determined by using the CORR procedure of SAS.

## 3. Results

### 3.1. Production Performance

The effects of monensin and BLFE on production performance during the last two weeks of the trial are shown in Table 1. As the significant interaction effects (*p* < 0.05) in Table 1 show, BLFE increased milk yield by 3.30 kg/d and feed efficiency (milk yield/DMI: 1.20 to 1.28) when added to the feed alone. No difference, however, was observed when BLFE was added to the feed together with monensin. The BLFE tended to increase milk protein content (*p* = 0.09), while monensin increased ECM by 2.45 kg/d (*p* = 0.02) and tended to increase milk fat content (*p* = 0.09), regardless of the presence or absence of the other feed additive in the diet. The improvements in milk fat content (4.22% to 4.48%) and ECM (4.04 kg/d) caused by monensin were significant in multiparous cows (*p* < 0.05), whereas they were negligible in primiparous cows (*p* > 0.10).

### 3.2. Alpha and Beta Diversity of Rumen and Fecal Microbial Communities

Table 2 presents the individual and interactive effects of R and BLFE on the alpha diversity indices, including the observed and predicted numbers of OTUs (Chao1 index), as well as the Shannon and Simpson indices, in rumen fluid and feces. Neither BLFE nor monensin significantly altered any of these alpha diversity parameters, either individually or through interaction (*p* > 0.10).

**Figure 1:**
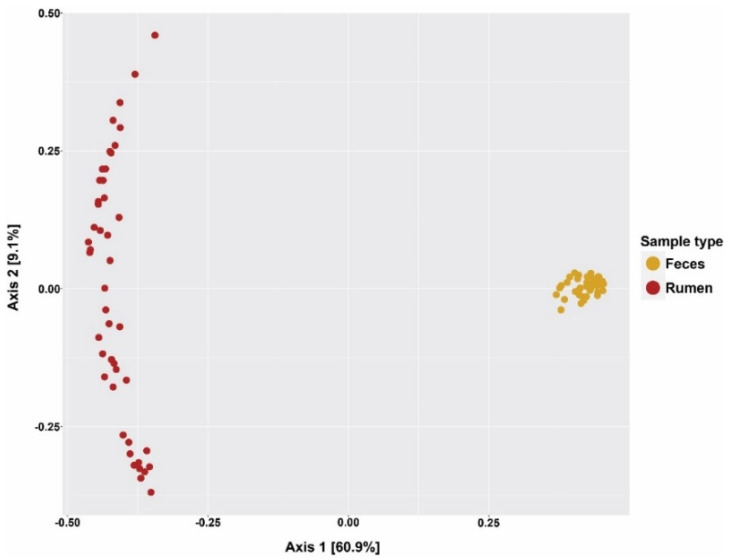
Principal coordinates analysis (PCoA) plot showing individual samples clustered according to categories of rumen fluid and feces.

Table 3 compares the differences in alpha diversity parameters between the rumen and feces microbiota communities when analyzed across all four treatment groups. Compared to the fecal microbiota, the number of observed OTUs and the number of predicted OTUs (Chao1 index) in the rumen microbiota increased by approximately 40%, indicating an improvement in microbiota richness (*p* < 0.01). Despite the lower levels of species richness, the Simpson index for the feces was higher than that of the rumen fluid (*p* < 0.01), suggesting a higher evenness in microbiota abundance in feces, relative to rumen fluid. Figure 1 presents a PCoA plot comparing the beta diversity of microbiota communities in the rumen and fecal samples across the treatments. The PCoA plot reveals two distinct clusters for rumen-fluid and fecal samples, indicating distinct differences in microbiota composition between the rumen and the lower gut. In alignment with the higher Simpson index, this plot shows a less diverse fecal microbiota composition compared to the rumen microbiota across samples. Furthermore, the PCoA plot analyses, which focused on samples stratified by treatment groups, revealed negligible effects of the treatments on the beta diversity for both rumen and fecal microbial communities. These negligible effects of the treatments were verified using a permutational multivariate analysis of variance (PERMANOVA) test, which again revealed no significant effects of the treatments in rumen-fluid or fecal samples (*p* > 0.10, Supplementary Table S1).

### 3.3. Kingdom and Phylum Abundance

Table 3 presents differences in kingdom and phylum abundances between rumen fluid and feces when analyzed across treatments. The abundances of the Bacterial and Archaeal kingdoms in rumen fluid and feces were similar, at 97.7% and 2.75%, respectively (*p* > 0.10). Fecal *Firmicutes* abundance was higher than that in rumen fluid (*p* < 0.01), whereas rumen *Bacteroidetes* abundance was higher than the fecal abundance (*p* < 0.01). Additionally, Table 3 provides the molar % of acetate and propionate, as well as the P: A, in rumen fluid and feces. Feces had a higher molar % of acetate, whereas propionate molar % was higher in rumen fluid, which led to higher P: A in rumen fluid than in feces (*p* < 0.01).

**Figure 2:**
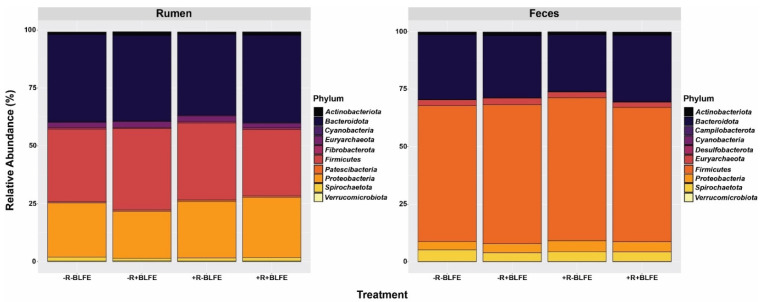
Relative abundances (%) of the 10 most abundant phyla in rumen fluid or feces of lactating cows receiving one of four dietary treatments: −R−BLFE (diet without both monensin and BLFE); −R+BLFE (diet with only BLFE), +R−BLFE (diet with only monensin); and +R+BLFE (diet with both monensin and BLFE).

Figure 2 presents the top 10 phyla in each of the rumen-fluid and feces samples by treatment group. The top three phyla in rumen fluid were *Firmicutes*, *Bacteroidetes*, and *Proteobacteria*, making up 36.40%, 31.72%, and 24.47% of the total rumen-fluid microbiota, respectively. In feces, *Firmicutes* were the most abundant, followed by *Bacteroidetes* and *Spirochaetota*, accounting for 60.07%, 27.10%, and 4.33% of the total OTUs, respectively. None of these top phyla showed a significant change in response to the dietary treatments (*p* > 0.10).

Table 4 summarizes the treatments’ effects on the relative abundances of the Bacteria and Archaea kingdoms, as well as the abundances of the *Firmicutes* and *Bacteroidetes* phyla and their ratio (F: B), in rumen fluid and feces, separately. Monensin and BLFE interactively modified *Firmicutes* abundance and F: B in rumen fluid (*p* < 0.10), such that BLFE tended to increase *Firmicutes* abundance and significantly increased F: B from 0.82 to 1.02 only with an absence of monensin in the diet. Additionally, the results indicated an interaction effect between monensin and parity relative to ruminal F: B, such that monensin increased and decreased F: B in primiparous and multiparous cows, respectively (*p* < 0.05). Monensin and BLFE did not affect the abundances of kingdoms and phyla in feces, either individually or interactively (*p* > 0.10).

**Figure 3:**
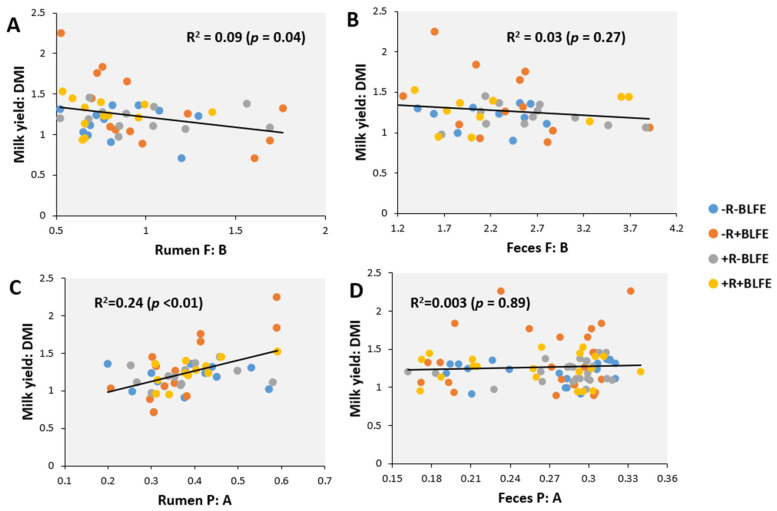
The relationships of feed efficiency (Milk yield/DMI) with propionate/acetate ratio (P: A) in rumen fluid (**A**) or feces (**B**) and the relative abundances comprising *Firmicutes*/*Bacteroidetes* (F: B) in rumen fluid (**C**) or feces (**D**).

Figure 3 presents the relationships of the F: B and P: A in rumen fluid or feces with feed efficiency (milk yield/DMI), analyzed using the data across treatments. These results showed a negative but weak relationship between ruminal F: B and feed efficiency (R^2^ = 0.09; *p* = 0.04, Figure 3A) and moderately positive relationship between ruminal P: A and feed efficiency (R^2^ = 0.24; *p* < 0.01, Figure 3C), while the relationships of fecal F: B and P: A were negligible (*p* > 0.10, Figure 3B,D).

### 3.4. Relative Abundances of Dominant Genera in Rumen Fluid and Feces

**Figure 4:**
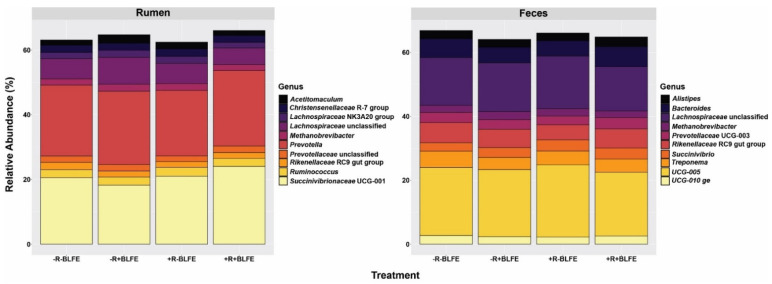
Relative abundances (%) of the 10 most abundant genera in rumen fluid and feces of lactating cows receiving one of four dietary treatments: −R−BLFE (diet without both monensin and BLFE); −R+BLFE (diet with only BLFE), +R−BLFE (diet with only monensin); and +R+BLFE (diet with both monensin and BLFE).

Figure 4 presents the relative abundances of the 10 most abundant rumen and fecal genera by dietary treatment. The top 10 genera in the rumen-fluid samples were *Prevotella*, *Succinivibrionceae_*UCG-001, unclassified_*Lachnospiraceae*, *Ruminococcus*, *Christensenellaceae_*R-7_group, *Lachnospiraceae_*NK3A20_group, *Methanobrevibacter*, *Acetitomaculum*, *Prevotellaceae_*UCG-004, and F082_ge. Among them, *Prevotella*, *Succinivibrionceae_*UCG-001, and unclassified*_Lachnospiraceae* were the most abundant, making up 21.98%, 21.86%, and 6.40% of the total OTUs, respectively. The top ten genera in the fecal samples were *Ruminococcaceae* UCG-005, unclassified*_Lachnospiraceae, Rikenellaceae-*RC9_gut_group, *Bacteroides*, *Treponema*, *Succinivibrio*, *Prevotellaceae_*UCG-003, *Alistipes*, UCG-010_ge, and *Methanobrevibacter*. The three most abundant fecal genera were *Ruminococcaceae* UCG-005 (21.20%), unclassified*_Lachnospiraceae* (15.19%), and *Rikenellaceae-*RC9_gut_group (5.66%).

### 3.5. Treatments’ Effects on Ruminal and Fecal Genera Abundances

Table 5 presents the relative abundances of genera in rumen fluid and feces that were significantly affected by monensin, BLFE, or their interaction. Of the 50 most abundant genera in rumen fluid, dietary treatments affected the abundances of only four genera (Table 5). The BLFE increased the abundances of *Bifidobacterium* and *Erysipelotrichaceae_*UCG-002 (*p* < 0.05), regardless of monensin in the diet. Monensin increased the abundance of *Oscillospirales_*ge (*p* = 0.02) but decreased the abundance of an unclassified *Clostridia* (*p* = 0.03). Monensin and BLFE modified the relative abundances of 12 genera among the 50 most abundant in feces, including *Bacteroides*, *Prevotella, Rikenellaceae*_RC9_gut_group, *Christensenellaceae*_R-7_group, Unclassified *Oscillospiraceae, Frisingicoccus*, *Parasutterella*, *Anaesporobacter*, *Lachnospiraceae*_UCG-001, *Monoglobus, Turicibacter*, and two uncultured genera (Table 5). The relative abundances of all of these genera, except unclassified_*Oscillospiraceae*, was affected by interactions between monensin and BLFE. The BLFE increased the abundance of Unclassified_*Oscillospiraceae* independent of monensin in the diet (*p* < 0.01). Supplementing BLFE alone increased *Prevotella* abundance (*p* = 0.04), but adding BLFE with monensin did not change it (*p* = 0.98). Moreover, BLFE or monensin alone decreased the abundance of *Christensenellaceae*_R-7_group (*p* < 0.05), while supplementing them together attenuated that decrease (Table 5).

### 3.6. Correlations Between Ruminal and Fecal Genera Abundances and Feed Efficiency

Table 6 presents the top genera in rumen fluid or feces that had statistically significant (*p* < 0.05) correlations with feed efficiency and P: A in the corresponding ecosystem. Table 6 also provides those correlations (*p* < 0.05) for genera affected by dietary treatments. Of the 20 most abundant genera in rumen fluid, the abundances of 10 had weak to moderate relationships with feed efficiency (|0.33| < r < |0.55|). Nine of those ten genera had negative correlations with both feed efficiency and rumen fluid P: A. The remaining genus, *Succinivibrionaceae_*UCG-001, had a moderately positive relationship with feed efficiency (r = 0.55, *p* < 0.01) and a strong positive correlation with rumen fluid P: A (r = 0.84, *p* < 0.01). Among the four genera in rumen fluid that were affected by dietary treatments, only the unclassified *Clostridia* abundance had a negative but weak correlation with milk yield/DMI (r = −0.30, *p* = 0.04). It also had a negative relationship with ruminal P: A, which, however, was stronger than that with feed efficiency (r = −0.66, *p* < 0.01).

Of the twenty most abundant genera in feces, only five (*Succinivibrio*, Unclassified_*Lachnospiraceae*, *Prevotella*, *Bacteroidales*_RF16_group_ge, and *Christensenellaceae*_R-7_group) had weak negative or positive relationships with feed efficiency, and their relationships with fecal P: A were negligible (*p* > 0.05, Table 6). Among the twelve fecal genera affected by dietary treatments (Table 5), the abundances of only four genera (*Prevotella*, *Christensenellaceae*_R-7_group, *Frisingicoccus*, and *Monoglobus*) were weakly related to feed efficiency. Their relationships with fecal P: A were also negligible (*p* > 0.05).

## 4. Discussion

Improving feed efficiency while maintaining or enhancing milk production is critical to achieving economic and environmental sustainability in dairy production. Manipulation of rumen microbiota composition, and thus, the fermentation profile, is a promising strategy for improving feed efficiency, one primarily achieved through microbial feed additives such as ionophores, prebiotics, and postbiotics [4,5,6,7,8,9,10,19]. Some research suggests that a considerable proportion of these feed additives can bypass the rumen and affect the composition of the lower-gut microbiota, which can also enhance feed efficiency [2,13]. To improve the current understanding of how gastrointestinal microbiota composition would response to these feed additives and how these responses would be related to the feed efficiency, in this study, the effects of a traditionally used ionophore feed additive, monensin, and a novel fermentation extract of *Bacillus licheniformis*, BLFE, on ruminal and fecal microbiota composition in dairy cows were assessed after feeding them for six weeks. It was hypothesized that monensin and BLFE would modulate gut microbiota compositions and, thereby, feed efficiency, in an additive fashion. Nevertheless, contrary to this hypothesis, the production data collected during the time the rumen and feces samples were taken revealed that each feed additive improved milk production performance or feed efficiency (milk yield/DMI) when fed alone, rather than in combination. These results align with the findings from the analysis of production data collected during the entire six weeks [26], suggesting that the timing of the rumen fluid and feces sample collection for the microbiome analyses was appropriate for capturing the inherent relationships between microbiota composition and feed efficiency. The overall microbial diversity, assessed with alpha and beta diversity analyses [37], however, indicated negligible changes in both rumen fluid and feces in response to the dietary treatments, suggesting the need to have a more in-depth investigation into specific compositional changes to explain the involvement of gut microbiota in the observed production and efficiency improvements. The alpha diversity in rumen fluid was of special interest, as a previous study has demonstrated a link between rumen-fluid alpha diversity and feed efficiency in dairy cows [2]. Furthermore, the literature highlights the potential for monensin to modulate ruminal microbiota species richness in goats and beef cattle fed forage-rich diets [38,39,40]. The unmodified alpha diversity of both the ruminal and fecal microbiota in this study, in contrast to those previous findings, suggests that the impacts of feed additives on gut microbiota may vary, depending on factors such as the host animal’s genetics and diet composition [41]. Nevertheless, the present study was designed primarily to assess the impacts of feed additives on production performance while minimizing the application of invasive sampling methods that would compromise the likelihood of accomplishing this objective. Including a periodic ruminal and fecal sampling schedule, which would also allow for determining the baseline composition of gut microbiota, along with increasing the sample size, could have more effectively captured the effects of the dietary treatments on gut microbiota diversity.

The present study’s results accurately represent the distinct differences between the ruminal and hindgut microbiota compositions, and these findings corroborate the notion that feces are not a suitable proxy for describing ruminal microbiota composition [42,43,44]. The observed increase in microbiota richness in the rumen is likely a result of the greater diversity among the nutritional substrates, diverse interactions among microbial communities, and the physical attributes of the rumen, such as ruminal contractions, which enhance interactions between microbial communities and their substrates [42,43]. Considering that *Firmicutes* and *Bacteroidetes* are mostly structural and non-structural carbohydrate fermenters, respectively [45], the observed increase in F: B in feces compared to rumen fluid reflects the expected reductions in the availability of non-structural carbohydrates (NSC), such as starch and sugars, relative to structural carbohydrates (SC), such as cellulose and hemicellulose, in the hindgut [46]. It is also important to note that the differences in F: B between these two ecosystems can be further influenced by the differences in the abundance of other phyla within them [47,48,49].

**Figure 5:**
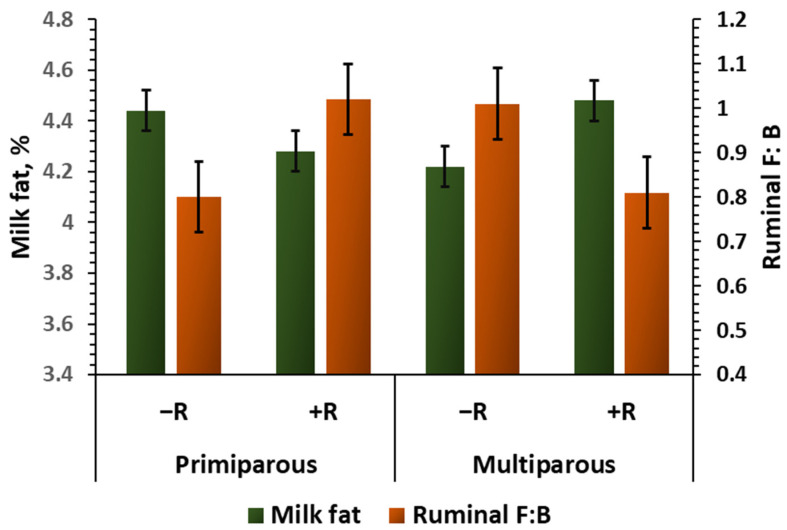
A graphical representation of the interactions (*p* < 0.05) between the presence or absence of monensin (+R and −R, respectively) in the diets of multiparous and primiparous cows relative to milk fat content (%) and the abundances comprising *Firmicutes/Bacteroidetes* in the rumen fluid (Ruminal F: B).

In support of the representativeness of the ruminal and fecal samples, the observed abundances of bacterial and archaeal kingdoms, as well as the top phyla identified in rumen fluid and feces, were consistent with the findings of previous studies [42,50,51]. Among the top phyla, monensin tended to suppress the abundance of *Firmicutes*, and thereby decreased F: B, in the rumen only in the presence of BLFE. Considering that many *Firmicutes* have Gram-positive-type cell membranes, this suppression is not entirely surprising, even though the reasons why this suppression was significant in the presence of BLFE remain unclear. Additionally, the results of the present study demonstrate, for the first time, an interaction between monensin and parity in the modulation of ruminal microbiota composition, such that monensin increased the ruminal F: B in primiparous cows, whereas it decreased this ratio in multiparous cows. While the underlying mechanisms of this interaction are unclear, the F: B reduction in multiparous cows aligns with the findings of McGarvey et al. [27]. Interestingly, this monensin × parity interaction effect on ruminal F: B was inversely related to the monensin × parity interaction effect on milk fat content (Figure 5), suggesting a negative relationship between ruminal fluid F: B and milk fat content, which aligns with the increased abundance of *Firmicutes* and the decreased abundance of *Bacteroidetes* observed in the rumen fluid from cows with induced milk-fat depression [52]. Relative changes in the abundances of ruminal *Firmicutes* and *Bacteroidetes* can vary depending on the diet composition, particularly the starch and unsaturated fatty acid contents [52]. Therefore, the impact of monensin on milk fat content could vary depending on these dietary factors as well.

**Figure 6:**
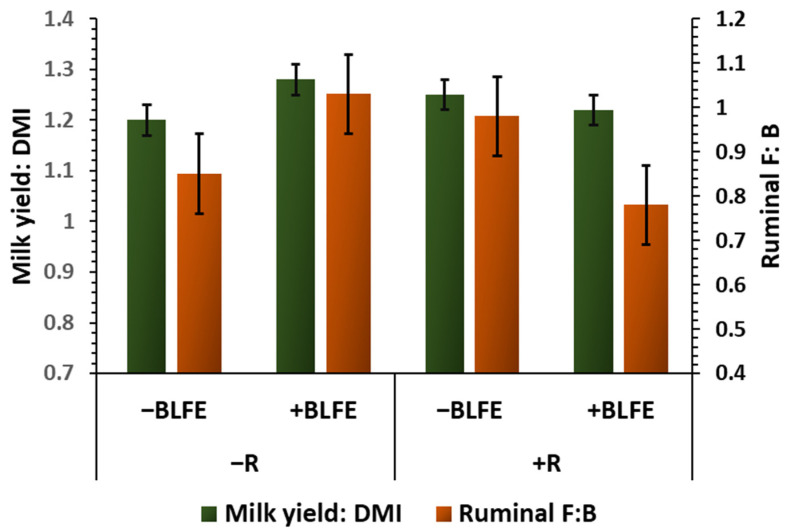
A graphical representation of the interactions (*p* < 0.05) between the presence or absence of monensin (+R and −R, respectively) and the presence or absence of BLFE (+BLFE and −BLFE, respectively) relative to feed efficiency (milk yield/DMI) and the abundances comprising *Firmicutes/Bacteroidetes* in rumen fluid (Ruminal F: B).

The observed negative relationship between ruminal F: B and feed efficiency is consistent with findings from Lopes et al. [53], who also identified a negative correlation between these two factors in beef cattle. However, the directions of the feed-efficiency response to dietary treatments do not align with this, suggesting that the relationship (r = −0.30) is not strong enough to reflect a direct cause-and-effect relationship. For example, the observed reduction in ruminal F: B caused by monensin supplemented to the diet with BLFE did not lead to an improvement in feed efficiency (+BLFE+R vs. +BLFE−R in Figure 6), and the improvement in feed efficiency attributed to feeding BLFE alone was associated with a trend toward an increase rather than a decrease in the F: B in rumen fluid (+BLFE−R vs. −BLFE−R in Figure 5). The negligible associations observed among fecal F: B, fecal P: A, and feed efficiency, as well as insignificant effects of the treatments on fecal F: B suggest a marginal involvement of the fecal microbiota composition and corresponding fermentation profile in modulating the feed-efficiency responses of dairy cows in this study.

Consistent with the literature [42], *Prevotella* and *Succinivibrionaceae_UCG-001* were the most abundant genera, accounting for approximately 50% of the total OTUs in the rumen fluid. However, both the rumen-fluid and fecal samples had higher abundances of *Lachnospiraceae* and *Methanobrevibacter* than those found in previous studies [42]. Considering the negative relationships between each of these microbiota taxa and feed-efficiency traits [54,55], their high abundances in this study may explain the lower feed efficiency (milk yield/DMI) of the current cow cohort (1.20 to 1.28, Table 1), compared to the expected feed efficiency of dairy cows in North America (1.5 to 1.6; [56]). The observed increase in ruminal *Bifidobacterium* abundance associated with BLFE could be beneficial in enhancing feed efficiency, as this bacterium has been shown to improve nutrient digestibility and the health status of farm animals [57,58]. The BLFE may boost the abundance of *Bifidobacterium* in the rumen by increasing starch digestibility through its amylase activity [59,60]. The other genus in the rumen that BLFE enhanced was *Erysipelotrichaceae_*UCG-002. Some research suggests that the growth of *Erysipelotrichaceae*_UCG-002 may also benefit from elevated starch digestibility [61,62]. Among the top genera in the rumen, monensin increased the abundances of a genus of order *Oscillospirales*, most of which are Gram-positive. Therefore, this observation may contradict the long-established action of monensin, which involves a suppression of growth in Gram-positive bacteria [63]. On the other hand, the observed decline in *Clostridia* abundance, as caused by monensin, supports this theory. Recent research, however, emphasizes that the impact of monensin on Gram-positive vs. Gram-negative bacteria is not always straightforward. For instance, Scharen et al. [18] studied the effects of monensin on rumen microbiota and concluded that the varying sensitivities of bacteria to monensin depend on the varying cell-wall composition and thickness, rather than on being Gram-negative or Gram-positive. Nevertheless, this unclassified *Clostridium* genus showed a negative correlation with ruminal P: A (r = −0.66, *p* < 0.01), suggesting that it could predominantly be acetate-producing. Furthermore, the current data demonstrate a positive relationship between the abundance of *Succinivibrionaceae* and propionate production in the rumen, highlighting positive implications of this relationship for feed efficiency, as supported by existing literature [64,65]. However, neither monensin nor BLFE, which increased ECM and feed efficiency, had an impact on the abundance of ruminal *Succinivibrionaceae*. These results underscore the complexity of the biology that connects the ruminal microbiota and their fermentation profiles with milk synthesis and its efficiencies. They also emphasize the need for robust experimental methods in capturing the influences of dietary interventions, such as feed additives, on this connection. Although the data indicate that fecal microbiota and their fermentation profiles have minimal associations with feed efficiency, it is essential to acknowledge that the composition of the lower-gut microbiota can still influence feed efficiency through mechanisms other than the contribution of their fermentation products to nutrient supply. For example, they may enhance immune function, which in turn improves the partitioning of nutrients towards milk production [66].

## 5. Conclusions

Dietary supplementation of monensin (14 mg/kg of DM) and a fermentation extract from *Bacillus licheniformis* (145 mg/kg of DM) interactively modified ruminal *Firmicutes*/*Bacteroidetes* (F: B) and feed efficiency (milk yield/DMI), such that the fermentation extract (BLFE) increased ruminal F: B and feed efficiency when fed alone rather than with monensin. Monensin interacted with parity to affect milk fat and ruminal F: B, such that it increased milk fat content and ruminal F: B in multiparous cows, while it decreased ruminal F: B and did not have an effect on milk fat content in primiparous cows. Monensin affected the abundances of two genera among the 50 most abundant in rumen fluid and increased fat-corrected milk yield, regardless of the presence of BLFE in the diet. Regardless of the presence of monensin in the diet, BLFE tended to increase milk protein and affected the abundances of two dominant ruminal genera. Monensin and BLFE did not modify fecal F: B individually or through interaction between them, and fecal F: B had a negligible relationship with feed efficiency. Overall, the study’s results demonstrated that monensin and BLFE supplementations can improve milk production performance and feed efficiency by modulating ruminal microbiota composition, and are more effective when added alone rather than in combination, in mid-lactation Holstein cows.

## Figures and Tables

**Table 1:** Treatments’ effects on production performance during the last two weeks of the study.

Variable	−R ^1^		+R		SEM	*p*-Value		
	−BLFE	+BLFE	−BLFE	+BLFE		R	BLFE	R × BLFE
DMI, kg/d	30.3	31.1	30.8	31.0	0.62	0.74	0.38	0.62
Milk yield, kg/d	35.4 ^b^	38.7 ^a^	38.0 ^ab^	37.3 ^ab^	0.63	0.51	0.05	<0.01
Milk yield/DMI ^2^	1.20 ^b^	1.28 ^a^	1.25 ^ab^	1.22 ^ab^	0.03	0.65	0.38	0.03
Milk protein, %	3.40	3.48	3.46	3.47	0.03	0.27	0.09	0.14
Milk fat, %	4.33	4.26	4.46	4.37	0.07	0.09	0.27	0.95
ECM, kg/d	40.4	41.9	44.1	43.1	1.02	0.02	0.75	0.23

^1^ −R (diet without monensin); +R (diet with monensin); −BLFE (diet without BLFE); +BLFE (diet with BLFE). ^2^ DMI = dry matter intake; ECM = energy-corrected milk. ^ab^ Different superscripts in a row indicate significantly different least-squares means (*p* < 0.05).

**Table 2:** Treatments’ effects on alpha diversity parameters in rumen-fluid and feces samples.

Parameter	−R ^1^		+R		SEM	*p*-Value		
	−BLFE	+BLFE	−BLFE	+BLFE		R	BLFE	R × BLFE
**Rumen fluid**								
Obs. OTU ^2^	2269	2174	2235	2123	105	0.68	0.32	0.94
Chao1	2937	2783	2907	2731	119	0.73	0.17	0.92
Shannon	5.36	5.34	5.33	5.20	0.21	0.68	0.72	0.80
Simpson	0.93	0.94	0.94	0.93	0.02	0.75	0.93	0.62
**Feces**								
Obs. OUT ^2^	1596	1542	1542	1596	37.4	0.31	0.74	0.28
Chao1	1972	1952	2006	2064	52.1	0.17	0.72	0.46
Shannon	5.35	5.28	5.35	5.40	0.05	0.28	0.80	0.23
Simpson	0.98	0.98	0.98	0.98	< 0.01	0.28	0.70	0.10

^1^ −R (diet without monensin); +R (diet with monensin); −BLFE (diet without BLFE); +BLFE (diet with BLFE).^2^ the number of observed OTUs.

**Table 3:** Alpha diversity parameters, kingdom and dominant phylum relative abundances (%), and volatile fatty acid molar percentages, in rumen fluid and feces.

Variable	Microbial Community		SEM	*p*-Value
	Rumen Fluid	Feces		
**Alpha diversity**				
Observed OTUs	2195	1599	39.4	<0.01
Chao 1	2837	2013	45.2	<0.01
Shannon	5.29	5.34	0.08	0.65
Simpson	0.93	0.98	0.01	<0.01
**Relative abundance, %**				
Kingdom				
Bacteria	97.7	97.7	0.14	0.15
Archaea	2.29	2.60	0.14	0.10
Phylum				
*Firmicutes* (F)	32.2	59.9	1.15	<0.01
*Bacteroidetes* (B)	36.7	27.0	0.90	<0.01
F: B	0.89	2.34	0.08	<0.01
**VFA, molar %**				
Acetate	61.8	67.9	0.63	<0.01
Propionate	23.9	18.1	0.59	<0.01
Propionate/acetate	0.39	0.27	0.01	<0.01

**Table 4:** Treatments’ effects on relative abundances of kingdoms and phyla (%) in rumen fluid and feces.

		–R ^1^		+R		SEM	*p*-Value		
		–BLFE	+BLFE	–BLFE	+BLFE		R	BLFE	R × BLFE
**Rumen Fluid**									
Kingdom abundance									
Bacteria		97.7	97.5	97.5	97.9	0.27	0.76	0.89	0.30
Archaea		2.20	2.46	2.41	2.09	0.27	078	0.91	0.18
Phylum abundance									
*Firmicutes*		31.1	36.2	33.1	28.5	2.67	0.28	0.93	0.07
*Bacteroidetes*		37.4	36.3	34.6	37.4	1.89	0.65	0.64	0.30
F: B ^2^		0.85 ^ab^	1.03 ^a^	0.98 ^ab^	0.78 ^b^	0.09	0.54	0.93	0.04
**Feces**									
Kingdom abundance									
Bacteria		97.4	97.3	97.4	97.6	0.29	0.54	0.78	0.64
Archaea		2.63	2.73	2.63	2.43	0.29	060	0.88	0.60
Phylum abundance									
*Firmicutes*		59.6	60.2	61.9	58.0	1.74	0.98	0.34	0.20
*Bacteroidetes*		27.8	27.3	24.8	29.0	1.69	0.71	0.29	0.16
F: B^2^		2.20	2.33	2.58	2.19	0.21	0.54	0.53	0.20

^1^ −R (without monensin); +R (with monensin); −BLFE (without BLFE); +BLFE (with BLFE). ^2^ F: B = *Firmicutes*/*Bacteroidetes* abundance; ^ab^ Different superscripts in a row indicate significantly different least-squares means (*p* < 0.05).

**Table 5:** Treatments’ effects on genera abundances in rumen fluid and feces.

Taxonomy (SILVA v138)	−R ^1^		+R		SEM	*p*-Value		
	−BLFE	+BLFE	−BLFE	+BLFE		R	BLFE	R × BLFE
**Rumen Fluid**								
*Bifidobacterium*	0.43	0.91	0.36	0.65	0.16	0.30	0.03	0.56
*Erysipelotrichaceae*_UCG-002	0.08	0.21	0.08	0.18	0.04	0.85	0.01	0.71
*Oscillospirales*_ge	0.32	0.36	0.42	0.38	0.03	0.02	0.99	0.19
unclassified*_Clostridia*	0.36	0.43	0.30	0.20	0.06	0.03	0.86	0.19
**Feces**								
*Bacteroides*	6.00 ^ab^	4.87 ^b^	4.82 ^b^	6.23 ^a^	0.48	0.86	0.77	0.01
*Prevotella*	1.19 ^b^	2.67 ^a^	2.43 ^ab^	1.17 ^b^	0.52	0.80	0.83	0.01
*Rikenellaceae*_RC9_gut_group	6.32 ^a^	5.31 ^ab^	4.72 ^b^	6.03 ^ab^	0.49	0.37	0.76	0.02
*Christensenellaceae*_R-7_group	1.66 ^ab^	1.35 ^ab^	1.28 ^b^	1.74 ^a^	0.14	0.99	0.57	0.01
Unclassified*_Oscillospiraceae*	0.94 ^a^	0.71 ^ab^	0.55 ^b^	0.94 ^a^	0.10	0.36	0.41	<0.01
*Frisingicoccus*	0.29 ^b^	0.46 ^a^	0.41 ^ab^	0.30 ^ab^	0.06	0.69	0.65	0.03
*Parasutterella*	0.30 ^a^	0.23 ^ab^	0.18 ^b^	0.30 ^a^	0.03	0.36	0.36	<0.01
*Anaerosporobacter*	0.29 ^ab^	0.42 ^a^	0.35 ^ab^	0.19 ^b^	0.07	0.19	0.84	0.03
*Lachnospiraceae*_UCG-001	0.14 ^b^	0.28 ^a^	0.24 ^ab^	0.19 ^ab^	0.04	0.98	0.27	0.02
*Monoglobus*	0.64 ^ab^	0.51 ^b^	0.48 ^b^	0.75 ^a^	0.10	0.68	0.47	0.04
*Turicibacter*	0.13 ^c^	0.31 ^a^	0.24 ^abc^	0.18 ^bc^	0.04	0.84	0.22	0.01
*Oscillibacter*	0.20	0.29	0.24	0.27	0.02	0.56	<0.01	0.10

^1^ −R (without monensin); +R (with monensin); −BLFE (without BLFE); +BLFE (with BLFE); ^abc^ Different superscripts in a row indicate significantly different least-squares means (*p* < 0.05).

**Table 6:** Pearson correlation coefficients and their *p*-values (within parentheses) for the relationships of the relative abundances of the top genera or the genera affected by dietary treatments in rumen fluid or feces with feed efficiency (milk yield/DMI) and the corresponding propionate-to-acetate ratio (P: A).

Taxonomy (SILVA v138)	Milk Yield/DMI	Rumen Fluid P: A
**Rumen fluid**		
**Among the 20 most abundant**		
*Succinivibrionaceae*_UCG-001	0.55 (<0.01)	0.84 (<0.01)
*Prevotellaceae*_UCG-004	−0.43 (<0.01)	−0.67 (<0.01)
*Lachnospiraceae*_NK3A20	−0.31 (0.04)	−0.40 (0.01)
*Succiniclasticum*	−0.34 (0.02)	−0.59 (<0.01)
*Acetitomaculum*	−0.43 (<0.01)	−0.37 (0.01)
*Bacteroidales* p-251-o5_ge	−0.47 (<0.01)	−0.60 (<0.01)
Unclassified_*Ruminococcaceae*	−0.45 (<0.01)	−0.62 (<0.01)
*Rikenellaceae*_RC9_gut_group	−0.44 (<0.01)	−0.79 (<0.01)
*Bacteroidales*_RF16_group_ge	−0.33 (0.02)	−0.54 (<0.01)
*Christensenellaceae_R-7_group*	−0.44 (<0.01)	−0.78 (<0.01)
**Among those affected by treatments (Table 5)**		
Unclassified*_Clostridia*	−0.30 (0.04)	−0.66 (<0.01)
**Feces**		
**Among the 20 most abundant**		
*Succinivibrio*	−0.35 (0.02)	−0.16 (0.32)
Unclassified_*Lachnospiraceae*	−0.31 (0.04)	−0.16 (0.30)
*Prevotella*	−0.37 (0.01)	−0.13 (0.39)
*Bacteroidales*_RF16_group_ge	0.36 (0.02)	0.29 (0.06)
*Christensenellaceae*_R-7_group	0.30 (0.04)	−0.12 (0.42)
**Among those affected by treatments (Table 5)**		
*Prevotella*	−0.37 (0.01)	−0.13 (0.30)
*Christensenellaceae*_R-7_group	0.30 (0.04)	−0.12 (0.42)
*Frisingicoccus*	−0.35 (0.02)	−0.16 (0.30)
*Monoglobus*	0.38 (0.01)	0.08 (0.65)

## Data Availability

All datasets collected and analyzed during the current study are available from the corresponding author on fair request.

## Supplementary Materials

The following supporting information can be downloaded at: https://www.mdpi.com/article/10.3390/ani15202980/s1, Table S1: The results of PERMANOVA analysis of the treatment effects on beta-diversity.
