# Supplementary material for: Effects of a Bacillus licheniformis Fermentation Extract and Monensin on the Rumen and Hindgut Microbiota Composition of Lactating Dairy Cows
Source: Animals (Basel). 2025 Oct 15;15(20):2980. doi: 10.3390/ani15202980 (PMC12560897; doi:10.3390/ani15202980)
Supplement: Supplementary file 1 [file animals-15-02980-s001.zip › animals-3924149-supplementary.pdf]

Supplementary Table 1. The results of PERMANOVA analysis of the treatment effects on beta-diversity

**PERMANOVA output for feces by treatment**					
Source	df	Sum of squares	R2	Pseudo-*F*	*P*
Treatment	3	0.39	0.07	1.15	0.18
Residual	43	4.90	0.93		
Total	46	5.29	1.00		
**PERMANOVA output for rumen fluid by treatment**					
Source	df	Sum of squares	R2	Pseudo-*F*	*P*
Treatment	3	0.25	0.05	0.69	0.80
Residual	44	5.25	0.95		
Total	47	5.50	1.00		
